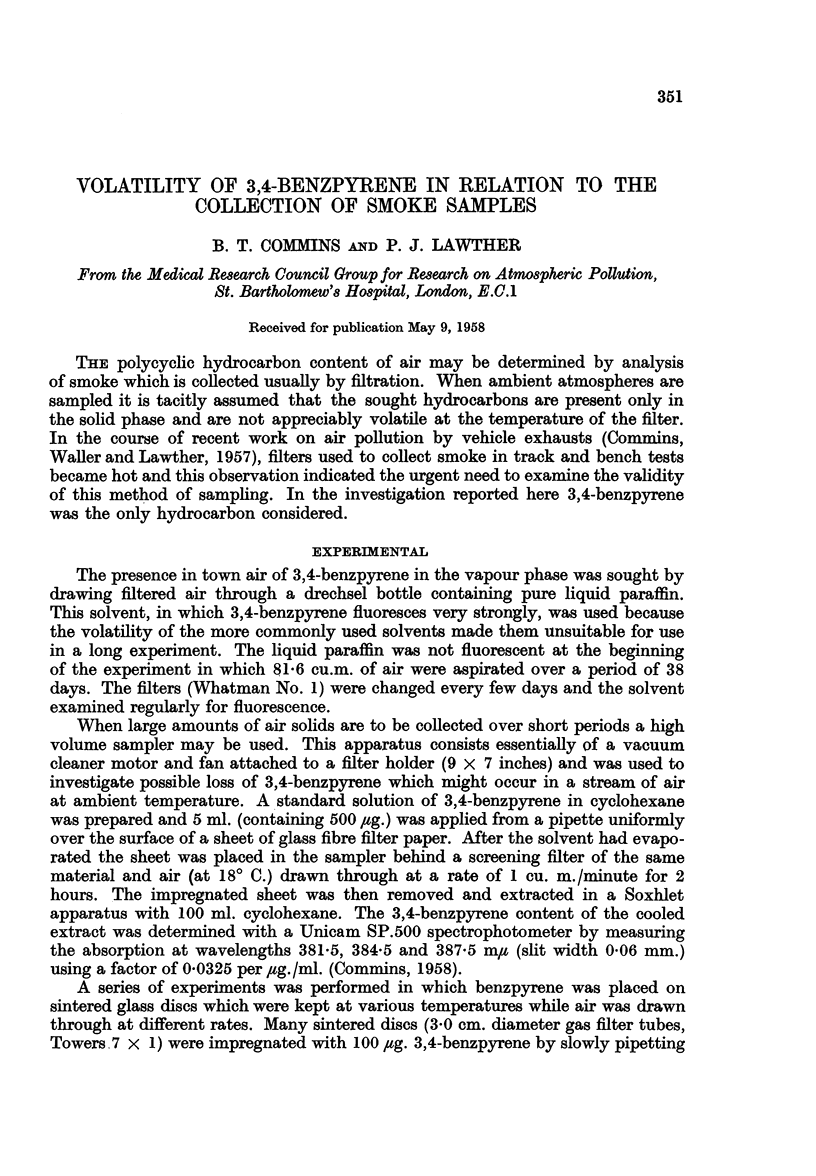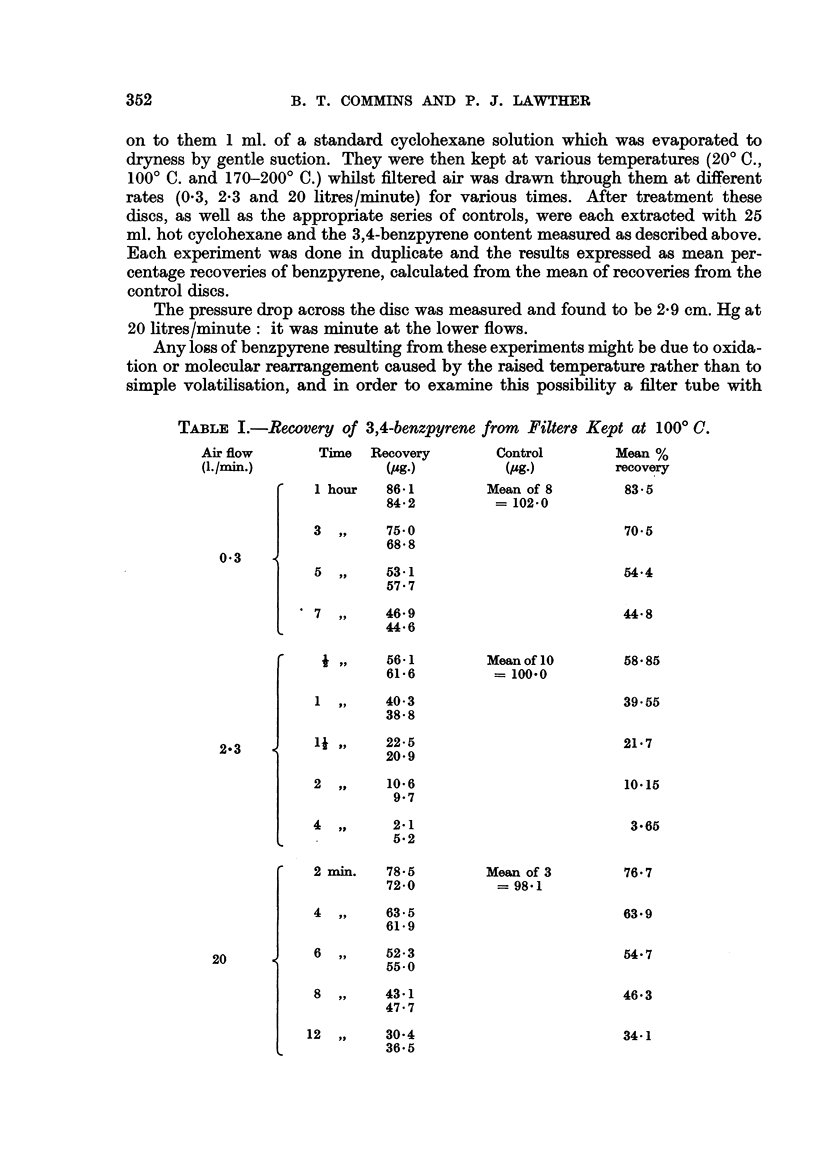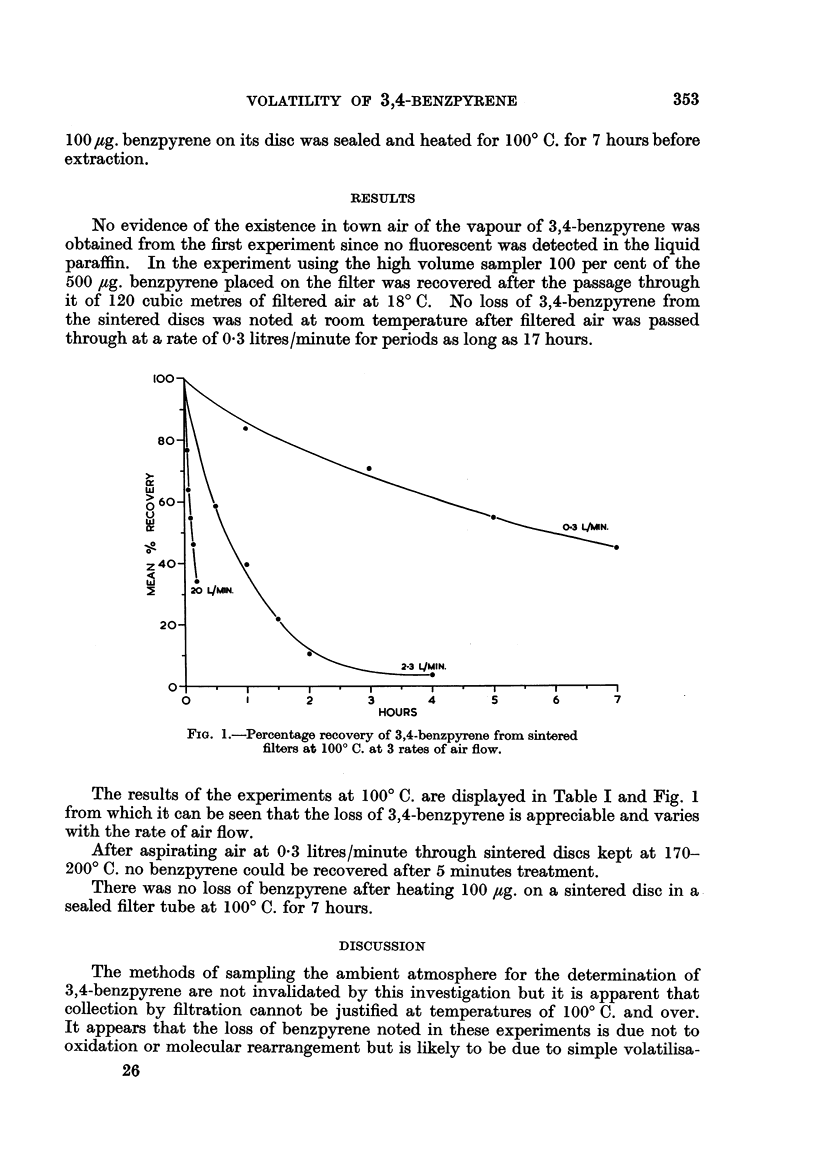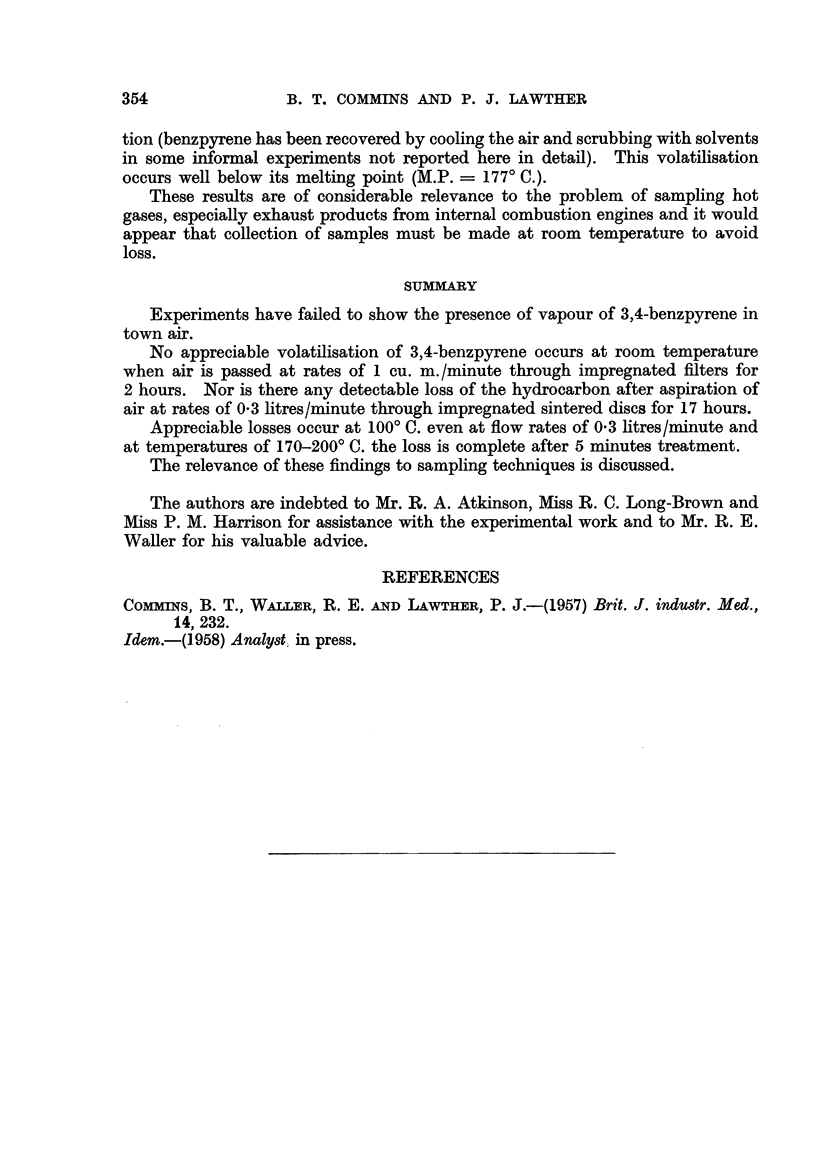# Volatility of 3,4-Benzpyrene in Relation to the Collection of Smoke Samples

**DOI:** 10.1038/bjc.1958.43

**Published:** 1958-09

**Authors:** B. T. Commins, P. J. Lawther


### VOLATILITY OF 3,4-BENZPYRENE IN RELATION TO THE COLLECTION OF SMOKE SAMPLES

B. T. COMMINS AND P. J. LAWTHER

*From the Medical Research Council Group for Research on Atmospheric Pollution, St. Bartholomew's Hospital, London, E.C.*1

Received for publication May 9, 1958

THE polycyclic hydrocarbon content of air may be determined by analysis of smoke which is collected usually by filtration. When ambient atmospheres are sampled it is tacitly assumed that the sought hydrocarbons are present only in the solid phase and are not appreciably volatile at the temperature of the filter. In the course of recent work on air pollution by vehicle exhausts (Commins, Waller and Lawther, 1957), filters used to collect smoke in track and bench tests became hot and this observation indicated the urgent need to examine the validity of this method of sampling. In the investigation reported here 3,4-benzpyrene was the only hydrocarbon considered.

### EXPERIMENTAL

The presence in town air of 3,4-benzpyrene in the vapour phase was sought by drawing filtered air through a drechsel bottle containing pure liquid paraffin. This solvent, in which 3,4-benzpyrene fluoresces very strongly, was used because the volatility of the more commonly used solvents made them unsuitable for use in a long experiment. The liquid paraffin was not fluorescent at the beginning of the experiment in which 81·6 cu.m. of air were aspirated over a period of 38 days. The filters (Whatman No. 1) were changed every few days and the solvent examined regularly for fluorescence.

When large amounts of air solids are to be collected over short periods a high volume sampler may be used. This apparatus consists essentially of a vacuum cleaner motor and fan attached to a filter holder (9 $\times$ 7 inches) and was used to investigate possible loss of 3,4-benzpyrene which might occur in a stream of air at ambient temperature. A standard solution of 3,4-benzpyrene in cyclohexane was prepared and 5 ml. (containing 500 $\mu$g.) was applied from a pipette uniformly over the surface of a sheet of glass fibre filter paper. After the solvent had evaporated the sheet was placed in the sampler behind a screening filter of the same material and air (at 18° C.) drawn through at a rate of 1 cu. m./minute for 2 hours. The impregnated sheet was then removed and extracted in a Soxhlet apparatus with 100 ml. cyclohexane. The 3,4-benzpyrene content of the cooled extract was determined with a Unicam SP.500 spectrophotometer by measuring the absorption at wavelengths 381·5, 384·5 and 387·5 m$\mu$ (slit width 0·06 mm.) using a factor of 0·0325 per $\mu$g./ml. (Commins, 1958).

A series of experiments was performed in which benzpyrene was placed on sintered glass discs which were kept at various temperatures while air was drawn through at different rates. Many sintered discs (3·0 cm. diameter gas filter tubes, Towers 7 $\times$ 1) were impregnated with 100 $\mu$g. 3,4-benzpyrene by slowly pipetting on to them 1 ml. of a standard cyclohexane solution which was evaporated to dryness by gentle suction. They were then kept at various temperatures (20° C., 100° C. and 170–200° C.) whilst filtered air was drawn through them at different rates (0·3, 2·3 and 20 litres/minute) for various times. After treatment these discs, as well as the appropriate series of controls, were each extracted with 25 ml. hot cyclohexane and the 3,4-benzpyrene content measured as described above. Each experiment was done in duplicate and the results expressed as mean percentage recoveries of benzpyrene, calculated from the mean of recoveries from the control discs.

The pressure drop across the disc was measured and found to be 2·9 cm. Hg at 20 litres/minute : it was minute at the lower flows.

Any loss of benzpyrene resulting from these experiments might be due to oxidation or molecular rearrangement caused by the raised temperature rather than to simple volatilisation, and in order to examine this possibility a filter tube with

TABLE I.—*Recovery of* 3,4-*benzpyrene from Filters Kept at* 100° *C.*

| Air flow (l./min.) | Time | Recovery (μg.) | Control (μg.) | Mean % recovery |
|---|---|---|---|---|
| 0·3 | 1 hour | 86·1 | Mean of 8 | 83·5 |
| | | 84·2 | = 102·0 | |
| | 3 „ | 75·0 | | 70·5 |
| | | 68·8 | | |
| | 5 „ | 53·1 | | 54·4 |
| | | 57·7 | | |
| | 7 „ | 46·9 | | 44·8 |
| | | 44·6 | | |
| 2·3 | ½ „ | 56·1 | Mean of 10 | 58·85 |
| | | 61·6 | = 100·0 | |
| | 1 „ | 40·3 | | 39·55 |
| | | 38·8 | | |
| | 1½ „ | 22·5 | | 21·7 |
| | | 20·9 | | |
| | 2 „ | 10·6 | | 10·15 |
| | | 9·7 | | |
| | 4 „ | 2·1 | | 3·65 |
| | | 5·2 | | |
| 20 | 2 min. | 78·5 | Mean of 3 | 76·7 |
| | | 72·0 | = 98·1 | |
| | 4 „ | 63·5 | | 63·9 |
| | | 61·9 | | |
| | 6 „ | 52·3 | | 54·7 |
| | | 55·0 | | |
| | 8 „ | 43·1 | | 46·3 |
| | | 47·7 | | |
| | 12 „ | 30·4 | | 34·1 |
| | | 36·5 | | |

100 $\mu$g. benzpyrene on its disc was sealed and heated for 100° C. for 7 hours before extraction.

### RESULTS

No evidence of the existence in town air of the vapour of 3,4-benzpyrene was obtained from the first experiment since no fluorescent was detected in the liquid paraffin. In the experiment using the high volume sampler 100 per cent of the 500 $\mu$g. benzpyrene placed on the filter was recovered after the passage through it of 120 cubic metres of filtered air at 18° C. No loss of 3,4-benzpyrene from the sintered discs was noted at room temperature after filtered air was passed through at a rate of 0·3 litres/minute for periods as long as 17 hours.

FIG. 1.—Percentage recovery of 3,4-benzpyrene from sintered filters at 100° C. at 3 rates of air flow.

The results of the experiments at 100° C. are displayed in Table I and Fig. 1 from which it can be seen that the loss of 3,4-benzpyrene is appreciable and varies with the rate of air flow.

After aspirating air at 0·3 litres/minute through sintered discs kept at 170–200° C. no benzpyrene could be recovered after 5 minutes treatment.

There was no loss of benzpyrene after heating 100 $\mu$g. on a sintered disc in a sealed filter tube at 100° C. for 7 hours.

### DISCUSSION

The methods of sampling the ambient atmosphere for the determination of 3,4-benzpyrene are not invalidated by this investigation but it is apparent that collection by filtration cannot be justified at temperatures of 100° C. and over. It appears that the loss of benzpyrene noted in these experiments is due not to oxidation or molecular rearrangement but is likely to be due to simple volatilisation (benzpyrene has been recovered by cooling the air and scrubbing with solvents in some informal experiments not reported here in detail). This volatilisation occurs well below its melting point (M.P. = 177° C.).

These results are of considerable relevance to the problem of sampling hot gases, especially exhaust products from internal combustion engines and it would appear that collection of samples must be made at room temperature to avoid loss.

SUMMARY

Experiments have failed to show the presence of vapour of 3,4-benzpyrene in town air.

No appreciable volatilisation of 3,4-benzpyrene occurs at room temperature when air is passed at rates of 1 cu. m./minute through impregnated filters for 2 hours. Nor is there any detectable loss of the hydrocarbon after aspiration of air at rates of 0·3 litres/minute through impregnated sintered discs for 17 hours.

Appreciable losses occur at 100° C. even at flow rates of 0·3 litres/minute and at temperatures of 170–200° C. the loss is complete after 5 minutes treatment.

The relevance of these findings to sampling techniques is discussed.

The authors are indebted to Mr. R. A. Atkinson, Miss R. C. Long-Brown and Miss P. M. Harrison for assistance with the experimental work and to Mr. R. E. Waller for his valuable advice.

### REFERENCES

Commins, B. T., Waller, R. E. and Lawther, P. J.—(1957) *Brit. J. industr. Med.*, **14**, 232.

*Idem.*—(1958) *Analyst*, in press.